# Microbial community development on model particles in the deep sulfidic waters of the Black Sea

**DOI:** 10.1111/1462-2920.15024

**Published:** 2020-04-30

**Authors:** Saara Suominen, Karlijn Doorenspleet, Jaap S. Sinninghe Damsté, Laura Villanueva

**Affiliations:** ^1^ Department of Marine Microbiology and Biogeochemistry NIOZ Royal Netherlands Institute for Sea Research and Utrecht University Den Burg The Netherlands; ^2^ Department of Earth Sciences, Faculty of Geosciences Utrecht University Utrecht, The Netherlands

## Abstract

Microorganisms attached to particles have been shown to be different from free‐living microbes and to display diverse metabolic activities. However, little is known about the ecotypes associated with particles and their substrate preference in anoxic marine waters. Here, we investigate the microbial community colonizing particles in the anoxic and sulfide‐rich waters of the Black Sea. We incubated beads coated with different substrates *in situ* at 1000 and 2000 m depth. After 6 h, the particle‐attached microbes were dominated by Gamma‐ and Alpha‐proteobacteria, and groups related to the phyla Latescibacteria, Bacteroidetes, Planctomycetes and Firmicutes, with substantial variation across the bead types, indicating that the attaching communities were selected by the substrate. Further laboratory incubations for 7 days suggested the presence of a community of highly specialized taxa. After incubation for 35 days, the microbial composition across all beads and depths was similar and primarily composed of putative sulfur cycling microbes. In addition to the major shared microbial groups, subdominant taxa on chitin and protein‐coated beads were detected pointing to specialized microbial degraders. These results highlight the role of particles as sites for attachment and biofilm formation, while the composition of organic matter defined a secondary part of the microbial community.

## Introduction

Microbial dynamics is of fundamental importance for the remineralisation of organic matter (OM) in marine environments, thereby contributing to long‐term carbon cycling processes (Azam and Malfatti, 2007; Jiao *et al*., 2010). At the surface, part of the OM aggregates to form particulate organic matter (POM) that is of a higher density and therefore sinks in the water column (Alldredge and Jackson, 1995). This POM, in addition to faecal material produced by zooplankton, is the main exporter of OM produced in surface waters by primary producers to the deep ocean and sediments (Herndl and Reinthaler, 2013), a process that is often referred to as the biological pump (Volk and Hoffert, 1985). Most of the POM undergoes degradation during settling in the water column, and only a small fraction of POM escapes remineralization and reaches the sediment, where it may, after subsequent break‐down, ultimately be buried (Burdige, 2007). Heterotrophic microbial activity is responsible for the degradation of POM as it sinks in the water column (Azam and Ammerman, 1984). The extent to how this process influences the water column microbial community dynamics remains unknown.

POM forms a physically separate microbial environment in aquatic ecosystems (Silver *et al*., 1978), as particulates harbour higher densities of organic carbon, nutrients and microbial cells than the surrounding water (Alldredge and Silver, 1988). Therefore, the ability to associate with particles is an important factor determining the microbial community composition and their activity (Grossart *et al*., 2006). Particle‐attached microbes are phylogenetically distinct from free‐living microbes (Salazar *et al*., 2015), but are consistently found across the same phylogenetic groups under widely varying biogeochemical conditions (DeLong *et al*., 1993; Fuchsman *et al*., 2011; Smith *et al*., 2013; Ganesh *et al*., 2014; Mestre *et al*., 2017; Bachmann *et al*., 2018; Suter *et al*., 2018). They include members of the Gammaproteobacteria, Deltaproteobacteria, Planctomycetes, Bacteroidetes and Verrucomicrobia. In agreement with this, the functional profile of particle‐attached microbes is distinguished mainly by the presence of genes related to motility, cellular adhesiveness and social interaction (Ganesh *et al*., 2014), as well as anaerobic metabolisms, that are induced in anoxic microniches formed inside POM during degradation (Smith *et al*., 2013). Community and metagenomic analyses of the microbial communities associated with particles indicate a predominantly heterotrophic lifestyle (Ganesh *et al*., 2014; Suter *et al*., 2018). However, the effect of the biochemical composition of POM on the composition of the particle‐attached microbial community and, hence, their lifestyles with respect to OM recycling remains largely unknown. So far, most studies into the identity of microbial communities degrading POM have focused on bulk material from size‐filtered suspended particulate matter (SPM) or sediment trap collected material, where the composition of the POM is unknown. A few studies exist that take into account the composition of POM. A study on natural POM across a continuum of particle types from aromatic‐rich terrestrial material in a freshwater system to carbohydrate‐rich particles in a marine system found that about 10% of the variation in the microbial community could be explained by differences in particle type (Zhang *et al*., 2016). In another study using model particles with known composition under oxic conditions, an initial community of microbes specialized on the hydrolysis of different polysaccharides, while a community of generalists rapidly took over the degradation process irrespective of the original polysaccharide (Enke *et al*., 2019).

Microbial communities are also strongly shaped by redox conditions in their environment and degradation of POM under anoxic conditions differs from that in oxic systems (Harvey *et al*., 1995; Van Mooy *et al*., 2002). A larger portion of OM escapes degradation in oxygen‐deficient waters (Karl and Knauer, 1991; Devol and Hartnett, 2001; Van Mooy *et al*., 2002; Roullier *et al*., 2014), but the exact reasons behind this phenomena are unknown (Keil *et al*., 2016). Despite this lower degree of mineralisation under anoxic conditions, rates of microbial POM degradation have repeatedly been found to be similar in oxic and anoxic conditions (Van Mooy *et al*., 2002; Cavan *et al*., 2017), though contrasting evidence also exists (Harvey *et al*., 1995). The main hypotheses explaining the lower degradation are therefore the lack of zooplankton grazing (Cavan *et al*., 2017), the refractory nature of OM on particles, or enhanced chemoautotrophic production of microbial biomass (Karl *et al*., 1984; Keil *et al*., 2016). In addition, in sulfidic sediments, sulfurization of OM may hinder its mineralisation (Sinninghe Damsté and de Leeuw, 1990). Although sulfurization of lipids typically occurs in the sediment (Werne *et al*., 2000; Sinninghe Damsté *et al*., 2007), sulfurization of carbohydrates may take place in the water column (Kok *et al*., 2000; Raven *et al*., 2016). Regarding the composition of OM, proteins seem to be a slightly preferred substrate over compounds with other biochemical composition for the mineralisation of OM under anoxic in comparison to oxic conditions (Harvey *et al*., 1995; Van Mooy *et al*., 2002; Engel *et al*., 2017). We hypothesise that the composition of POM also in anoxic conditions has a major impact on the ability of the microbial community to degrade it, and hence possibly also the microbial community attached to it.

In this study, we aimed to assess the identity of the particle‐attached and POM‐degrading microbial community in the anoxic and sulfidic waters of the Black Sea, where the strong stratification leads to a depletion of oxygen in deeper waters and, consequently, a specialized microbial community (Vetriani *et al*., 2003; Fuchsman *et al*., 2011). We used magnetic beads coated with specific organic substrates as models of marine particles as previously applied in oxic water incubations (Datta *et al*., 2016; Enke *et al*., 2019). To assess the development of particle‐attached microbial populations with time, we used both short‐term *in situ* incubations at 1000 and 2000 m depth, as well as longer lasting on deck incubations. By using different organic substrates, our aim was to assess how the composition of POM affects the microbial community composition under sulfidic conditions, a topic not previously studied but essential for our understanding of biogeochemical cycling of carbon in these types of environments.

## Results

### Physicochemical conditions and incubation experiments

**Fig. 1:**
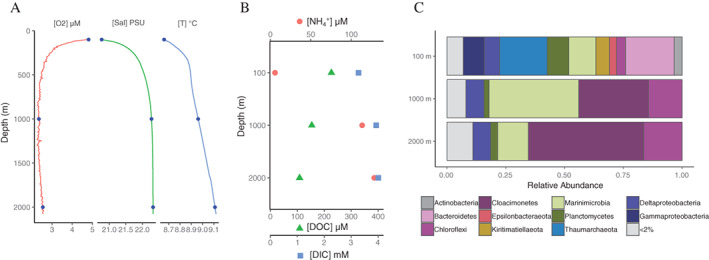
Initial water column conditions and the microbial communities at the three sampling depths 100, 1000 and 2000 m depth. A. Physical measurements across the total water column by CTD. B. Chemical analyses at the three sampling depths, DIC; dissolved inorganic carbon and DOC; dissolved organic carbon. C. Microbial community composition in suspended particulate matter (SPM) at the three sampling depths expressed as the relative abundance of 16S rRNA gene sequences. Phylum‐level taxonomy is shown for all except Proteobacteria were we show class‐level taxonomy. [Color figure can be viewed at wileyonlinelibrary.com]

At the time of sampling, oxygen was depleted from 300 μM at 30 m to 6 μM at 85 m marking the surface oxic layer. In this zone salinity was on average 19.1 ± 0.7 ppm, while temperature was high at the surface (26°C at 4 m depth) but quickly decreased reaching 9°C at 30 m (Fig. 1A). We did not measure inorganic nitrogen species across the whole water column, but according to measurements at the same site made in 2013 (Sollai *et al*., 2019), there was a maximum in the nitrate concentration at 70–80 m (~2.5 μM) and a nitrite peak at 85 m (0.04 μM). The suboxic zone, containing neither oxygen or sulfide in considerable amounts, was defined at 75–115 m, and the remaining water column was anoxic and contained sulfide (Sollai *et al*., 2019). This sulfidic zone was characterized by an accumulation of sulfide from 0.9 μM in the suboxic zone to approximately 400 μM at 2000 m. An increase in ammonium concentrations from 0.24 μM at 21 m to 96 μM at 2000 m and dissolved inorganic carbon (DIC) from 3.1 mM at 21 m to 4.0 mM at 2000 m was measured during our sampling campaign (Fig. 1B). DOC concentrations varied across the water column but were 225 ± 8 μM at the surface (21 m) and across the sulfidic zone were higher at 600–1200 m (160 ± 17 μM) than at greater depths 1300–2000 m (94 ± 8 μM) (Fig. 1B).

*In situ* incubations with beads coated with specific organic substrates were performed at 1000 and 2000 m for a total of 6 h. Two categories of beads were tested; magnetic cores with covalently bound pure chitin and magnetic cores with an agarose matrix coated with peptidoglycan, fatty acids, cellulose or without coating. In addition to *in situ* incubations, we performed incubations with these magnetic beads (as well as protein‐coated beads) in bottles on deck of the research vessel with water from the sulfidic (1000 and 2000 m) but also from the suboxic (100 m) redox zones with a longer time frame (7 and 35 days). For these sampled waters there were only minor differences in salinity (20.1 at 100 m; 22.3 at 1000 and 2000 m; Fig. 1A) between the sampling depths. A previous study at the same station (Sollai *et al*., 2019) detected no sulfide at 100 m, while at 1000 and 2000 m sulfide concentrations approached 400 μM (350 and 400 μM at 1000 and 2000 m respectively). Other major differences between the waters used for incubation studies were in the ammonium concentration, which increased substantially with depth, i.e. from 4.3 μM at 100 m to 85 μM at 1000 m, and to 96 μM at 2000 m (Fig. 1B). DIC concentrations also increased with depth from 3.3 mM at 100 m to 3.9 mM at 1000 m, and 4.0 mM at 2000 m (Fig. 1B). Nitrate was present at 100 m (1.6 μM), but not detectable in the deep waters. Nitrite concentrations were low at 100 m, while phosphate concentrations were stable and high across the depths sampled here (average of the three sampling depths 7.6 ± 0.3 μM). After 7 days of bottle incubations, DIC concentrations decreased compared with the original water column concentrations, while between 7 and 35 days of the incubations nitrite, nitrate and DIC concentrations increased (Fig. S1).

### Microbial community composition of SPM

The total microbial community of the SPM collected with a 0.3 μm‐pore‐size glass fibre filter at the three depths used for experimental incubations (100, 1000 and 2000 m) was analysed by 16S rRNA gene amplicon sequencing. The microbial community in the suboxic zone at 100 m was mainly composed of Bacteroidetes (20.7% of total 16S rRNA gene abundance) from the class Chlorobia (15.6%), Thaumarchaeota (20.2%), Proteobacteria (17.1%), Marinimicrobia (11.5%) and Planctomycetes (9.1%) (Fig. 1C). The main orders of Proteobacteria were Ectothiorhodospirales (6.3%) from Gammaproteobacteria (9.0%) and the order Desulfobacterales (4.2%) from the Deltaproteobacteria (6.5%). In the sulfidic waters (1000 and 2000 m), the community of the SPM was substantially different and dominated by operational taxonomic units (OTUs) from the uncultured microbial phyla Cloacimonetes (29.7% and 49.1% relative abundance at 1000 and 2000 m respectively), Marinimicrobia (38.1% and 13.0%) and Chloroflexi (14.3% and 16.3%), mainly from the class Anaerolineae (11.2% and 11.8%). Deltaproteobacteria (7.7% and 7.5%) dominated the Proteobacteria (8.1% and 7.9%), with members of the orders Desulfobacterales (3.5% and 4.5%) and Desulfarculales (3.6% and 2.6%) as the most abundant (Fig. 1C).

### Composition of the microbial community attached to beads during *in situ* incubations

**Fig. 2:**
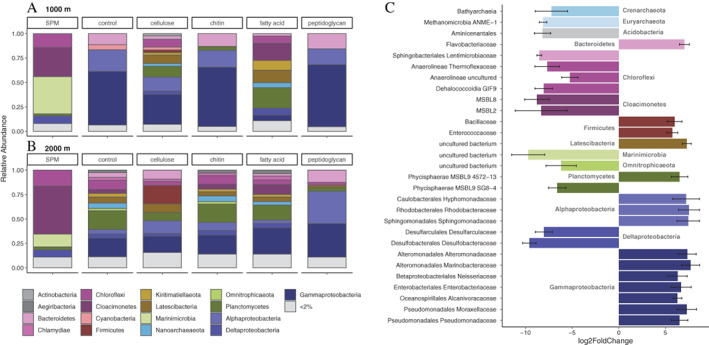
The microbial community developed in *in situ* incubations and its relation to the community in suspended particulate matter (SPM). A,B. Microbial community composition as the relative abundance of 16S rRNA gene sequences of substrate‐coated beads after *in situ* incubations for 6 h at 1000 m (A) and at 2000 m depth (B).Taxonomic affiliations shown at the phylum or Class level for Proteobacterial classes. C. Differentially abundant taxa at Family level compared between all *in situ* bead samples and SPM, combined from both 1000 and 2000 m depth samples. Bars show mean log_2_ fold change across all OTUs belonging to the taxa, and the colours are the same as in panels A and B. [Color figure can be viewed at wileyonlinelibrary.com]

We used pouches of nylon mesh fabric to incubate the beads *in situ* in the deep, sulfidic waters of the Black Sea (Fig. S2) for 6 h. All bead types except protein were incubated *in situ* (this included beads of pure chitin, pure agarose, or agarose coated with cellulose, fatty acids or peptidoglycan). This experimental design was aimed at directly observing local communities that readily attach to the various particles under *in situ* environmental conditions. The bead microbial communities were variable, but all of the samples differed strongly from the water column microbial communities (Fig. 2A and B). Variation across bead types was different at 1000 and 2000 m. At 1000 m, the microbial communities on control (agarose beads without additional coating), chitin and peptidoglycan beads were predominantly composed of sequences attributed to Gammaproteobacteria (55.3%, 58.5%, 63.1% relative abundance of the 16S rRNA gene respectively), Alphaproteobacteria (22.5%, 17.6%, 17.2%) and Bacteroidetes (10.7%, 14.2%, 15.1%). In addition, control beads had a minor abundance of cyanobacterial 16S rRNA gene sequences (3.8%), as also observed for Planctomycetes 16S rRNA gene sequences (4.8%) in chitin beads. Cellulose beads had also a high abundance of sequences attributed to Gamma‐ (29.5%) and Alpha‐proteobacteria (13.6%). In addition, the rest of the community on cellulose beads and the community on fatty acid‐coated beads were highly diverse, with major contribution from sequences affiliated to Planctomycetes (11.4% on cellulose beads and 21% on fatty acid beads), Latescibacteria (9% and 12.8%), Kirimatillaeota (2.5% and 10.1%), Nanoarchaeota (3.1% and 5.1%) and Chloroflexi (7.9% and 7.5%). The fatty acid‐coated beads also contained sequences affiliated to Cloacimonetes in considerable relative abundance (17.4%).

At 2000 m, the peptidoglycan beads had a community with sequences predominantly attributed to Gammaproteobacteria (34.4%), Alphaproteobacteria (33.1%) and Bacteroidetes (12.4%). The control, chitin and fatty acid beads had a diverse community composed mainly of Gammaproteobacteria (20.0% on control beads, 19.1% on chitin beads and 25.9% fatty acid‐coated beads), Planctomycetes (18.7%, 18.8%, 15.6%), Chloroflexi (10.4%, 9.2%, 4.8%), Cloacimonetes (4.0%, 5.0%, 10.7%), Nanoarchaeota (5.2%, 5.7%, 3.6%) and Latescibacteria (5.3%, 4.3%, 4.8%). The cellulose beads at 2000 m were dominated by Gamma‐ (17.3%) and Alpha‐proteobacteria (13.4%), Firmicutes (17.9%), Bacteroidetes (9.4%), Planctomycetes (8.3%) and Latescibacteria (8.9%).

The taxonomic groups found to be preferentially attached to the particles were determined by analysing the OTUs between SPM and all bead communities using a Deseq2 analysis (Love *et al*., 2014; McMurdie and Holmes, 2014), which calculates the statistically significant log_2_ of fold change between relative abundances in the different sample groups (Fig. 2C; see experimental procedures for details). The OTUs with a significantly different relative abundance are shown grouped at the Family level in Fig. 2C. Microbial taxa enriched on the beads belonged to the diverse families of the Gammaproteobacteria, Alphaproteobacteria, Planctomycetes, Firmicutes and Actinobacteria among others (Fig. 2C). The taxa that were more common in the water column SPM were mainly from families belonging to the Deltaproteobacteria, Cloacimonetes and Chloroflexi.

The community attached to the beads after incubation *in situ* differed significantly from that of the water column SPM (PERMANOVA *R* ^2^ = 0.116, *p* = 0.008). This is also shown with the non‐metric multidimensional scaling (NMDS) analysis obtained using Bray–Curtis distances of the various microbial communities (Fig. 4A), revealing that the composition of the SPM from 1000 and 2000 m is more similar than of any of the bead microbial communities. Duplicate incubation experiments were similar to each other for each bead type (Fig. 4A). The bead type explained about 25% of variability of the communities in beads incubations (PERMANOVA *R* ^2^ = 0.25, *p* = 0.001), while depth explained 10% (*R* ^2^ = 0.1, *p* = 0.001) (Table S1).

### Development in the composition of microbial communities on beads during bottle incubations

**Fig. 3:**
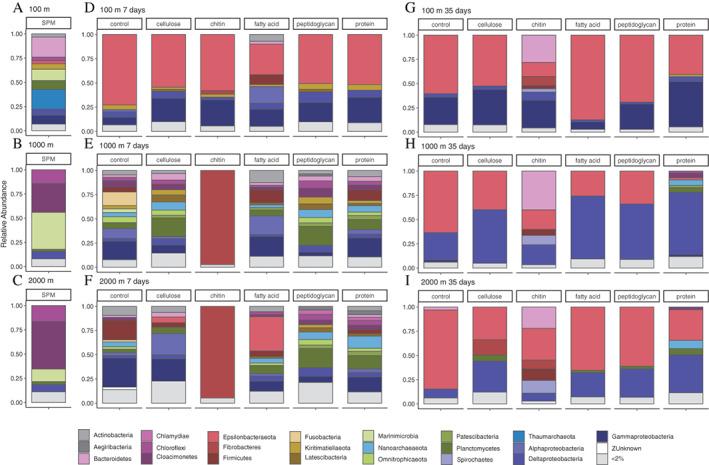
Microbial community composition of bottle incubations matter at 100, 1000 and 2000 m depth. The relative abundance of 16S rRNA gene sequences are shown for all samples at Phylum or Class level (for Proteobacteria), with triplicate experiments combined. A–C. The community composition of beads after incubation in bottles for 7 days and (D–F) after incubation for 35 days. [Color figure can be viewed at wileyonlinelibrary.com]

To test for changes in the microbial community attached to particles on a longer timeframe, we conducted bottle incubations on deck with the same magnetic beads used in the *in situ* incubations with collected sulfidic water (both from 1000 and 2000 m). In addition, to compare these results to water from another redox zone, we performed the same experiments with water from the suboxic zone (100 m). In the sulfidic water incubations, the microbial community developed on the chitin beads was clearly distinct from all other bead types and developed a community dominated by Chitinovibrionales from Fibrobacteres (95.8%) at day 7 (Fig. 3E–F), and a community with subdominant populations of Bacteroidetes (31%), Spirochaetes (11.4%) and Firmicutes (9.1%) at day 35 (Fig. 3H–I). Across all other remaining bead types, the most abundant microbial community members at 7 days in sulfidic waters were affiliated to the orders Gamma‐ (15.9% on average across all samples except chitin beads) and Alpha‐proteobacteria (8.8%) and phyla Planctomycetes (10.5%), Firmicutes (6.6%), Actinobacteria (6.1%), Nanoarchaeota (5.7%), as well as Epsilonbacteraeota (5.3%) (Fig. 3E–F). These taxa were further classified into the orders Planctomycetes Phycisphaerae (6.4%) Nanoarchaeota Woesearchaeia (4.7%) and Epsilonbacteraeota Campylobacteria (4.6%) as well as the classes Pseudomonadales (5.1%) and Rhizobiales (5.8%) from Gamma‐ and Alpha‐proteobacteria respectively. After 35 days, a community dominated by Epsilonbacteraeota Campylobacterales (40.3%) had developed with the second most dominant groups from Deltaproteobacteria at 1000 and 2000 m (49.6% and 23.8% respectively) (Fig. 3H–I).

The microbial communities that developed in suboxic water (100 m) incubations after 7 days of incubation were different from those in the sulfidic incubations (Fig. 3). In the suboxic water incubations, there was an enrichment of Epsilonbacteraeota (51.7%) *Sulfurimonas* (33.1%) and *Arcobacter* (17.7%), as well as Gammaproteobacteria (20.9%) Arenicellales (9.4%) and Deltaproteobacteria (6.7%) order Desulfobacterales (4.9%) (Fig. 3D). The community did not change substantially after 35 days of incubation with a dominance of the Epsilonbacteraeota (54.1%) genera *Arcobacter* (37.6%) and *Sulfurimonas* (15.9%) as well as Gammaproteobacteria (29%) mainly from the orders Oceanospirillales (12.6%) and Alteromonadales (9.0%).

**Fig. 4:**
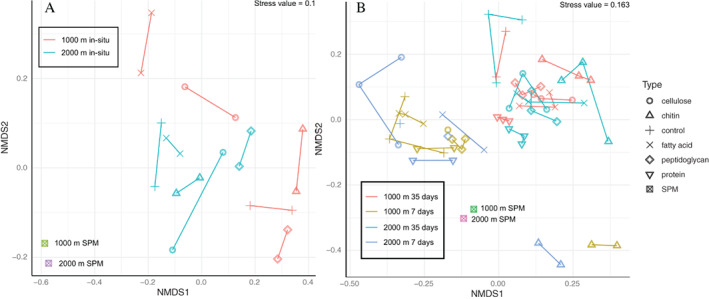
A NMDS ordination of Bray–Curtis distances of microbial communities formed on beads at bottle incubations. A. Bead incubations from *in situ* experiments, and (B) Bead incubations from 1000 and 2000 m depth water bottle incubations. Shapes differ by bead type. [Color figure can be viewed at wileyonlinelibrary.com]

For the sulfidic bottle incubations, an NMDS analysis using Bray–Curtis distances of the microbial communities revealed clustering by the duration of the incubations to samples taken at day 7 and at day 35 (Fig. 4B). As seen above for the *in situ* experiments (Fig. 2A), the SPM samples were more closely related to each other than to the bead samples, which had a variable community but were clustered together. The depth of water collection did not affect clustering, while within the samples taken at day 7 and day 35 there was some variation based on replicate incubations of different bead types (Fig. 4B). Specifically, replicates of chitin, protein and control beads were more closely related to each than the remaining bead types. In total, the bead type explained about 16% of variability in the data (PERMANOVA *R* ^2^ = 0.16, *p* = 0.001), and the duration of the incubations about 9% (PERMANOVA *R* ^2^ = 0.09, *p* = 0.001). The interaction between timepoint and type explained 12% (PERMANOVA R^2^ = 0.12, *p* = 0.001), and depth only explained 2% of variability (PERMANOVA *R* ^2^ = 0.02, *p* = 0.001). Because of the low contribution of depth to the variability, we considered samples from 1000 and 2000 m as replicates when analysing taxa that were significantly different between bead type incubations. The NMDS analysis showed that for suboxic water incubations sampling points 7 and 35 days, the differences between communities at different bead types was not enough to make a reliable ordination analysis (stress = 0.201). Bead type explained about 20% of variability (PERMANOVA *R* ^2^ = 0.20, *p* = 0.001), while time explained approximately 5% of variability (PERMANOVA *R* ^2^ = 0.05, *p* = 0.002).

We analysed the effect of the bead type on the microbial community by comparing communities from the separate bead types to each of the other bead types that were incubated. These pairwise PERMANOVA analyses found significant differences across bead types from incubations at day 7 and day 35 with sulfidic waters (Table 1). No significant differences associated to bead types were found in incubations with suboxic waters (i.e. 100 m depth), possibly due to having only three replicates of each treatment (compared with the six replicates by combining the results of the 1000 and 2000 m incubations). The tested beads fell into two categories; magnetic cores with covalently bound pure chitin or protein and magnetic cores with an agarose matrix coated with peptidoglycan, fatty acids, cellulose or without coating (control). Microbial communities on chitin and protein beads were different from agarose‐containing bead types at both sampling points (Table 1). In addition, on day 7 peptidoglycan beads showed a community composition significantly different from other bead types except of those coated with cellulose, and at day 35 all bead types were significantly different from the control beads.

**Table 1:** Pairwise PERMANOVA analysis comparing microbial communities on bead types at incubation sampling points day 7 and day 35.

	Day 7 communities sulfidic depths					Day 35 communities sulfidic depths				
Pairs	F‐Model	*R* ^2^	*p*‐value	*p* adj	Sig	F‐Model	*R* ^2^	*p*‐value	*p* adj	Sig
Protein vs. peptidoglycan	2.32	0.28	0.031	0.048	.	3.68	0.27	0.004	0.005	*
Protein vs. control	1.91	0.24	0.030	0.048	.	3.43	0.28	0.006	0.008	*
Protein vs. chitin	3.48	0.37	0.034	0.048	.	3.53	0.26	0.002	0.005	*
Protein vs. cellulose	1.67	0.22	0.030	0.048	.	2.62	0.21	0.003	0.005	*
Protein vs. fatty acid	1.78	0.20	0.006	0.045	.	2.70	0.21	0.003	0.005	*
Peptidoglycan vs. control	1.64	0.21	0.030	0.048	.	2.49	0.22	0.003	0.005	*
Peptidoglycan vs. chitin	3.62	0.38	0.021	0.048	.	2.33	0.19	0.003	0.005	*
Peptidoglycan vs. cellulose	1.62	0.21	0.063	0.079		1.37	0.12	0.057	0.066	
Peptidoglycan vs. fatty acid	1.80	0.20	0.024	0.048	.	1.27	0.11	0.065	0.070	
Control vs. chitin	2.38	0.28	0.026	0.048	.	2.05	0.19	0.002	0.005	*
Control vs. cellulose	1.07	0.15	0.301	0.323		1.87	0.17	0.004	0.005	*
Control vs. fatty acid	1.09	0.14	0.243	0.280		1.96	0.18	0.004	0.005	*
Chitin vs. cellulose	2.15	0.26	0.035	0.048	.	1.94	0.16	0.004	0.005	*
Chitin vs. fatty acid	2.50	0.26	0.006	0.045	.	1.87	0.16	0.004	0.005	*
Cellulose vs. fatty acid	0.95	0.12	0.552	0.552		0.97	0.09	0.447	0.447	

Comparisons across sulfidic sampling depths at 1000 and 2000 m. The Benjamini–Hochberg method was used to adjust acquired *p*‐values for multiple comparisons (*p* adj). Significance (sig) <0.05 shown with a point and <0.01 with a star.

**Fig. 5:**
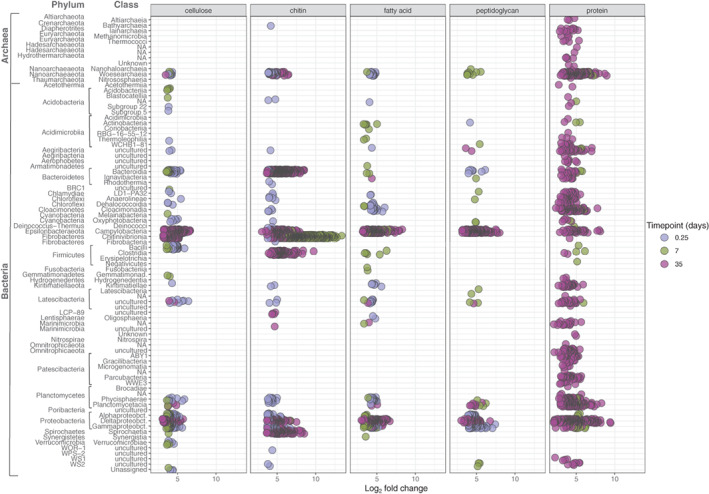
Microbial taxa that were differentially abundant from control (uncoated agarose beads) across the different bead types and sampling points from 1000 and 2000 m depth water incubations. Each point represents an individual OTU that was significantly enriched in a specific bead type compared with the control beads. Colour represents the different sampling points with 0.25 depicting microbial communities at *in situ* 6 h bead incubations. OTUs grouped at the Class level. Log_2_ fold change determined with a Deseq2 analysis. [Color figure can be viewed at wileyonlinelibrary.com]

To further analyse which microbial taxa differed in abundance because of the substrate coating the magnetic beads, we analysed the community members that were differentially abundant on each bead type compared with uncoated agarose beads with a Deseq2 differential abundance analysis (Fig. 5, see experimental procedures for details) (Love *et al*., 2014; McMurdie and Holmes, 2014). The analysis identifies OTUs that have a significantly higher abundance in treatment versus control samples, while normalizing for sample size differences and accounting for variance across replicates (examples of these differences in raw counts in Fig. S3). Therefore, this analysis was performed to determine if there were taxonomic groups specifically induced by the additional substrate on the beads, while not taking into account the overall relative abundance in the total community. At day 7 for the protein‐coated beads, taxonomic groups that had most OTUs that were significantly more abundant in protein versus uncoated (control) agarose beads were affiliated to the phyla Nanoarchaeota (17 OTUs), Proteobacteria (16 OTUs), Planctomycetes (eight OTUs) and Cloacimonetes (six OTUs). While for peptidoglycan‐coated beads, the most differentially abundant OTUs were from the phyla Nanoarchaeota (nine OTUs), Planctomycetes (four OTUs), Latescibacteria (four OTUs), WS2 (three OTUs) and Cyanobacteria (two OTUs). Despite an enrichment of a few dominant taxa in the community in most samples at day 35, significant differences were found in the microbial community composition related to the original substrate type. In chitin beads, diverse Bacteroidetes (425 OTUs) were significantly more abundant than in agarose beads; mainly from the orders Bacteroidales (303 OTUs) and Flavobacteriales (104 OTUs). In addition, members of the Spirochaetes (166 OTUs), Firmicutes Clostridiales (134 OTUs) and Fibrobacteres Chitinovibrionales (100 OTUs) were significantly more abundant. In protein‐coated beads, there was a more diverse group of microorganisms than in the control agarose beads (without coating), belonging to the phyla Proteobacteria (267 OTUs) class Deltaproteobacteria (253 OTUs), Planctomycetes (186 OTUs) classes Planctomycetacia (114 OTUs) and Phycisphaerae (68 OTUs), Nanoarchaeota (159 OTUs) class Woesearchaea (157 OTUs), diverse Patescibacteria (89 OTUs) mainly class Parcubacteria (39 OTUs) and Cloacimonetes (89 OTUs). The control uncoated agarose beads had relatively more Arcobacteraceae (236 OTUs) from Epsilonbacteraeota compared with all other bead types.

## Discussion

Microbial communities are of fundamental importance for marine carbon cycling and play a vital role in the degradation and remineralization of POM. In the Black Sea, suboxic microbial communities associated with SPM retained on 30 μm pore‐size filters have been found to be different from the free‐living fraction (Fuchsman *et al*., 2011). In a similar environment in the Cariaco basin the microbial community retained on 2.7 μm pore‐size filters were defined as organotrophic, and varying depending on the redox conditions (Suter *et al*., 2018). However, no comparison has thus far been made between specifically the POM‐associated community between suboxic and the anoxic basin in the Black Sea. In addition, in the mentioned studies, sample collection by filtration collected a bulk particulate fraction, that could not be separated by particle type and may also (partially) retain free‐living organisms. Particle composition also appears to be affecting microbial degradation rates (Van Mooy *et al*., 2002) but no study has been made on the effect of different carbon substrates on the POM‐associated microbial communities in anoxic waters. Furthermore, although changes of POM‐attached microbial communities with time has been suggested (Datta *et al*., 2016), this is thus far not shown specifically for anoxic POM‐attached communities, or in *in situ* conditions.

In this study, we designed and tested a novel *in situ* incubation approach to study particle‐associated microbial communities colonizing beads coated with different organic substrates. By using *in situ* incubated nylon mesh pouches that allow the free movement of microbes, but no release of substrate particles, we were able to determine the first attachment of microbes to particulate matter under *in situ* conditions, including *in situ* hydrostatic pressure. This experimental approach aims to bridge the gap between *in situ* conditions versus laboratory conditions, where most of such studies have been previously conducted. Even with careful recovery of anoxic marine waters, one of the main challenges with studying these communities remains with the changes induced by experimental methodology (Stewart *et al*., 2012; Torres‐Beltrán *et al*., 2019). Laboratory incubations allow the possibility for longer incubation times often required in energy‐limited environments like anoxic waters, but inherent factors like hydrostatic pressure or natural mixing cannot be mimicked. The observations from bottle incubations are thus difficult to connect to local conditions directly, but still can be used for the purpose to generate testable hypotheses that may shed light on particle‐attached microbial community present in anoxic marine waters.

### Microbial *in situ* attachment to model particles in deep sulfidic waters

Microbial communities readily attaching to particles in sulfidic waters were studied *in situ* with model magnetic beads in the Black Sea. The first particle colonizers were similar across all beads and both tested depths and showed considerable differences to the composition of the microbial community of SPM at these depths (Fig. 1). The most common groups among the first colonizers were Altermonadales (Gammaproteobacteria), Rhodobacterales (Alphaproteobacteria) and Flavobacteriales (Bacteroidetes), as well as heterotrophic Planctomycetes. These microbes have been previously associated with a particle‐attached lifestyle in studies on incubations conducted with particles collected by sediment traps in the North Pacific Subtropical Gyre (Fontanez *et al*., 2015) as well as in oxic abyssal waters in the open ocean (Boeuf *et al*., 2019). These microbes have also been isolated from model marine particles (Datta *et al*., 2016; Enke *et al*., 2019) and are known for their rapid particle attachment and biofilm formation capabilities (DeLong *et al*., 1993; Lee *et al*., 2008; Doghri *et al*., 2015). The presence of the same taxonomic groups of microbes attached to the particles in the anoxic and sulfidic waters of the Black Sea is surprising, and possibly confirms the notion of a taxonomically distinct microbial ecotype whose predominant lifestyle is particle‐attached (Salazar *et al*., 2015). Their ability to attach to particles also in a highly reduced environment indicates that this ecotype is present independent of redox conditions. Another possible reason explaining the similar taxonomic composition of the particle‐attached microbial community of our study in sulfidic conditions compared with other studies in oxic waters is the importance of anaerobic metabolisms within POM. Genes indicative of anaerobic metabolisms are found on particles present in oxic conditions, probably due to anoxic microniches formed inside the particles (Smith *et al*., 2013), and could therefore indicate that the same taxa could survive also with surrounding sulfidic waters. A third possible explanation is that substrate utilization may be relatively independent on oxygen availability in marine environments. Common taxa incorporating glucose in surface waters have been shown to do this in both oxic and anoxic conditions (Alonso and Pernthaler, 2005). Taken together, here we conclude that water column redox conditions do not appear to affect the POM‐associated microbial community to a significant extent. On the other hand, a few taxonomic groups seem to be specific to oxygen‐depleted conditions; the relatively enriched Latescibacteria (formerly candidate division WS3) and Omnitrophica (formerly candidate division OP3) are more abundant in sulfidic environments as they have been previously found highly enriched in SPM specifically in the oxygen‐depleted waters of the Cariaco basin (Suter *et al*., 2018) and the Black Sea (Fuchsman *et al*., 2011). This confirms the niche of these groups as microbes actively attaching to particles in oxygen‐depleted conditions.

As defined by the PERMANOVA analysis, about 25% of variation in the microbial community composition of the beads during the *in situ* incubation could be assigned to the type of bead coating (Fig. 4A). At 1000 m, the microbial composition of the fatty acid‐coated beads was considerably different, possibly reflecting the lack of a sugar backbone in the coating substrate that may be preferred by copiotrophic organisms, that are adapted to proliferate quickly when a suitable substrate becomes available. In fact, the most common taxa on the beads, Gamma‐ and Alpha‐proteobacteria, as well as Bacteroidetes, have been previously recognized as rapid degraders of sugars in anoxic marine incubations (Alonso and Pernthaler, 2005). Additionally, polysaccharides are known to be rapidly degraded in anoxic marine systems (Arnosti *et al*., 1994), which could be an indication of their importance as a carbon source for heterotrophic prokaryotes in these systems. At 2000 m depth, the situation was different, with the composition of the microbial community of the fatty acid‐coated beads similar to the control (uncoated agarose) and cellulose‐coated beads, and in general a lower abundance of the Proteobacterial classes. It may be that at 2000 m the microbial community is less ‘ready’ to degrade labile substrates, resulting in a slower recruitment of the copiotrophic microbes. The peptidoglycan‐coated beads were the only bead type with considerable abundances of Proteobacterial classes also at 2000 m. This could reflect the microbial benefit of readiness to degrade peptidoglycan also in deep waters. It is possible that the capacity to attach to peptidoglycan would aid these microorganisms in finding labile detrital OM also in the deep sulfidic water column. In addition, cellulose‐coated beads attracted a different microbial community from that observed in the control beads at both *in situ* incubation depths. Specifically, the phylum Firmicutes was found in considerable abundance (>1%) only on the cellulose beads incubated under *in situ* conditions. This could be an indication of abilities of this phylum to attach to cellulose substrates and specificity to degrade it. Taxa belonging to the Clostridium genus of the phylum Firmicutes are commonly recognized as important degraders of cellulose under thermophilic and anaerobic conditions (Schwarz, 2001; Ji *et al*., 2012), which supports our finding.

In our study, ship‐time limitations restricted our *in situ* incubation times, but when possible, the use of *in situ* methodologies, like the one used here, for longer timeframes and combined with (meta)omic analyses could further advance the understanding of microbial functionality in hard‐to‐reach environments. Nevertheless, despite the 6 h‐*in situ* incubation time frame used in our study, the variation across the microbial communities across bead types showed that the local community was rapidly induced and selected by the available labile substrate sources even in deep sulfidic waters at 2000 m.

### Succession of the microbial community on model particles in bottle incubations

A shift in the microbial community was detected on model particles in the bottle incubations with water from sulfidic depths (1000 and 2000 m depth). After 7 days of incubation, the various bead types had developed variable microbial communities different from that existing in the original SPM. An enrichment of Chitinovibrionales (>90% relative abundance of the community) developed on chitin beads that was highly specific to the substrate type, attesting that in the sulfidic waters this was an appropriate timeframe to stimulate active degraders of chitin. However, no such specific enrichment was found for the other bead types. Instead, a variable community formed across the different types of model particles. Excluding the chitin beads, the microbes that were enriched belonged to the taxa defined by rapid attachment as observed in the *in situ* incubations; i.e. Gamma‐ and Alpha‐proteobacteria as well as Planctomycetes, Omnitrophica, Latescibacteria and Cloacimonetes. The taxonomic composition of the microbial community was therefore similar to that of the beads that were incubated *in situ*.

On the other hand, at 7 days of incubation in suboxic water and at 35 days in both sulfidic and suboxic waters a relatively simple microbial community developed, which was predominantly composed of sulfur‐oxidizing lineages of Epsilonbacteraeota, Campylobacterales and Gammaproteobacteria (suboxic water) or Deltaproteobacteria (sulfidic water, both at 1000 and 2000 m; Fig. 3D, G–I). The earlier establishment of this community in the incubation with suboxic waters could suggest that the development of a particle‐attached niche is faster at suboxic conditions, where the redox potential is higher, and more terminal electron acceptors are presumably available for sulfide oxidation. This coincides with the depth range where Epsilonbacteraeota are found naturally at their highest numbers (Grote *et al*., 2008; Henkel *et al*., 2019) also showing their efficient use of the traces of sulfide. The development of this community at sulfidic conditions after a month's incubation could indicate a mature particle‐attached community, where the substrate has been depleted and is possibly not influencing the microbial community composition any longer to a large extent. A remarkably similar highly specialized microbial community has previously been reported from sinking particulate matter in oxic conditions in the North Pacific Subtropical Gyre (Fontanez *et al*., 2015). In that study, the particle‐attached community of sinking particulate matter was compared by either poisoning the sample immediately after collection or allowing the trapped sinking particulate matter to incubate un‐poisoned in the sediment trap. While the un‐poisoned sinking particulate matter had a community of organisms related to Gammaproteobacteria and Bacteroidetes similar to the particle‐attached niche observed in other studies of size‐fractioned SPM, the poisoned samples contained specialized groups of putative sulfur‐oxidizing microbes (Fontanez *et al*., 2015), similar to the ones observed in our study on the beads after on‐deck incubation. This could indicate that larger, sinking particles in fact act as a surface for biofilm formation of a specialized ecotype in marine water columns, rather than being defined by the organic material present on the particles. These taxa have been detected as initial members of biofilms in sulfide‐rich hydrothermal fluids (Gulmann *et al*., 2015), indicating that they benefit from a close association in biofilms. A similar community has also been detected and metagenomically characterized in sinking particles in the oxic abyssal open ocean (Boeuf *et al*., 2019), providing further evidence that particles reaching the deep sea and that are collected by sediment traps contain a specialized microbial community compared with SPM. It is possible that after the labile organic material on particles is degraded, the minerals present on sinking particles (Hedges *et al*., 2001) or the sulfurization of POM in the sulfidic water column (Raven *et al*., 2016) are in fact shielding parts of the organic material from microbial degradation. Taken together these results indicate that heterotrophic activity on sinking particulate matter decreases with depth in the water column, and this has to do with the mineral content of the particulate matter itself. The putative chemoautotrophic communities enriched in our experiments could therefore directly reflect the processes that happen in natural sinking particles across depth in marine water columns. In addition to the specialization to sinking particulate matter, these taxonomic groups of bacteria were also previously detected in the higher size fraction of SPM in the Black Sea (Fuchsman *et al*., 2011, 2012a), though mainly at shallower depths, as well as in connection to a nitrate‐rich plume (Fuchsman *et al*., 2012b). Epsilonproteobacteria are known as active denitrifiers at redox gradients in the marine pelagic environments (Campbell *et al*., 2006; Bruckner *et al*., 2013; Waite *et al*., 2017). An increase of nitrite and nitrate concentrations with time in our bottle incubations indicates that nitrogen metabolisms were active and may, at least in part, be responsible for the activity of sulfide‐oxidizers on the beads tested in our study. However, at the depths where we obtained the water used for our incubation experiments nitrate is depleted, so these metabolisms can only be driven by trace amounts of nitrogen species present or arising from local recycling of matter. Dinitrogen losses from OMZs are directly connected to OM concentrations (Ward *et al*., 2008; Kalvelage *et al*., 2013), and denitrification from nitrogen inside organic particles has been hypothesized as the source of nitrogen loss (Fuchsman *et al*., 2019). The development of a possibly nitrate‐reducing community and active nitrogen cycling in our particles confirms these findings. Recently, an isolate from the Black Sea belonging to the *Sulfurimonas* genus has also been characterized as a sulfide‐oxidizing manganese reducer (Henkel *et al*., 2019), making it plausible that reduction of metals could also play a part as an electron sink for this chemoautotrophic population. The magnetite particles forming the core of our magnetic beads contain a mixture of Fe(II) and Fe(III), which perhaps could be utilized by known iron‐reducing bacterial lineages (Byrne *et al*., 2016). Iron‐rich particles are common in the suboxic zone of the Black Sea, forming up to 30% of all particles (Schulz‐Vogt *et al*., 2019), and are part of cycles of phosphorus and manganese with complex dynamics (Dellwig *et al*., 2010). In sulfidic environments, dissolved iron oxides react rapidly with sulfide forming iron sulfide minerals (Canfield and Berner, 1987), which could also increase the conductivity of these particles (Malvankar *et al*., 2015). Due to the complex interactions of metals in anoxic environments, the full extent the iron oxides that are available for microbial reduction in the model particles used here remains, however, unknown.

Similar to what was previously detected by Fuchsman *et al*. (2011), another abundant microbial group in the experiments with sulfidic waters after 35 days was Desulfuromonadales. These microbes are possibly connected to sulfate‐ or elemental sulfur‐reducing microbial groups, indicating a complex activity connected to multiple oxidation states of sulfuric compounds on the bead particles. Particularly, these results highlight once more the possibility that particles act predominantly as locations of chemoautotrophic metabolism. The contribution of microbial chemoautotrophic production to enhanced particle fluxes in oxygen deficient marine environments has previously been demonstrated for the Arabian Sea OMZ (Keil *et al*., 2016), the anoxic Cariaco basin (Taylor *et al*., 2001), as well as the Black Sea (Karl and Knauer, 1991; Sorokin *et al*., 1995; Çoban‐Yildiz *et al*., 2006). In addition to extensive evidence from OMZs globally, the finding of similar organisms in natural environments (Fuchsman *et al*., 2011; Fontanez *et al*., 2015; Boeuf *et al*., 2019) indicates that chemoautotrophic microbial populations could be influencing the dynamics and fluxes of natural particles when oxygen becomes depleted.

### Effect of OM type on microbial community composition

In both *in situ* and in bottle incubations, the composition of the microbial community was determined for magnetic beads coated with different organic substrate types; pure chitin, protein and agarose (control), as well as agarose with covalently attached peptidoglycan, cellulose and fatty acids. The effect of bead type at different sampling times and depths was not always the main defining factor of the community, but underlying patterns in the microbial community were separated by substrate type. Specifically, the microbial community composition of the chitin and protein‐containing beads differed clearly from the agarose‐containing beads in bottle incubations.

Throughout the incubation experiments, the microbial community present on the chitin‐coated beads were the most different from the community existing in the remaining beads. Chitin is one of the most abundant biopolymers in aquatic ecosystems and is produced in the marine environment by crustaceans and diatoms (Jeuniaux and Voss‐Foucart, 1991; Beier and Bertilsson, 2013). Therefore, it is a substrate type that is possibly available naturally in a highly enriched particulate form. Chitin was also hypothesized to be one of the most rapidly degraded substrates when comparing biochemically different phytoplankton substrates under anoxic and oxic conditions (Harvey *et al*., 1995). In addition, chitin‐degrading enzymes were significantly enriched in sinking particles in a metagenomics study (Fontanez *et al*., 2015). The members of the Chitinovibrionia class that were enriched at day 7 belong to the small phylum of Fibrobacteres. Known members of this phylum are almost exclusively anaerobic obligatory fermenters lacking electron transport chains and specialized to the degradation of polymers (Rahman *et al*., 2016). The genome of an isolated species from the same genus was categorized as an alkaliphilic organism capable of using cell‐bound chitinases (Sorokin *et al*., 2014), making it possible that it benefits from a particle‐attached lifestyle in close association with its substrate. At day 35, the microbial community detected on chitin beads was still significantly different from all other bead types, mainly because of a diverse subdominant microbial community. Taxa from the phyla Bacteroidetes, Spirochaetes, Firmicutes and diverse Deltaproteobacteria were more abundant in chitin beads compared with control beads (Fig. 5). This community is therefore most probably degrading secondary products derived from chitin degradation. Surprisingly, this was the case also in the incubations with suboxic water, despite of the fact that the initial Chitinovibrionia degrader was not detected at the earlier sampling point in suboxic conditions. This is either an indication that the initial degrader was missed at these sampling points, that subdominant populations in the suboxic incubations like members of Gammaproteobacteria were degrading the chitin matrix, or that some of the Epsilonbacteraeota could be acting as heterotrophic degraders under nitrate‐replete conditions.

For the remaining bead types (i.e. excluding chitin), the microbial communities at day 7 were variable across all substrate types. The fact that the microbial community of the beads, including those coated with protein, resembled that of the agarose‐containing beads (control) indicates that at this stage the microbial community on particles was predominantly being defined by attachment rather than degradation of the polysaccharide or coating substrate. Contrastingly, at day 35 of incubation there were more differences in the underlying communities of the remaining bead types that could be connected to substrate type, despite the dominant community members being connected to chemoautotrophic metabolisms discussed in the preceding section. Interestingly, protein‐coated beads had developed a diverse subdominant community, indicating that degradation of the proteinaceous substrate was an active process. These include diverse groups of organisms with small genomes from the candidate phyla radiation of bacteria Patescibacteria and Nanoarchaeota (Nelson and Stegen, 2015; Liu *et al*., 2018), as well as members of Chloroflexi and Actinobacteria (Fig. 5). The role of organisms with small genomes in the environment is unknown, and inferring activity from genomics is difficult because of the lack of many essential metabolic pathways and abundance of unknown genes. However, both Nanoarchaeota and Patescibacteria seem to share a metabolic strategy and evolutionary history (Castelle *et al*., 2018), and have been connected to protein degradation in the Black sea water column with a DNA‐SIP study recently (Suominen *et al*., 2020). Similarly, Actinobacteria have been previously connected to protein degradation in both oxic (Orsi *et al*., 2016) and sulfidic (Suominen *et al*., 2020) conditions, providing further evidence that this subdominant community was induced by the provided substrate.

Though the agarose beads were coated with molecules with different structural components (peptidoglycan, cellulose and fatty acids), it seems that the inevitably lower concentration of the substrate molecules was possibly slowing and disguising the development of a degrading microbial community, as pairwise PERMANOVA analyses did not consistently reveal differences between these bead types (Table 1). Some minor differences could be detected in the community composition, when looking at the differentially abundant organisms (Fig. 5). For example, in cellulose‐coated beads at day 35, there was a population of Chitinovibrio most probably linked to the degradation of the cellulose backbone. For the remaining bead types (fatty acid, cellulose and peptidoglycan), they did not differ from control at day 7, nor did they differ from each other at day 35. However, at day 35, control agarose beads were significantly different from all the other agarose bead types coated with an additional substrate (peptidoglycan, cellulose or fatty acids). It is possible that the coating substrates induced communities reliant on an external carbon source, while the community on uncoated agarose beads was more reliant on autotrophic carbon fixation. A higher abundance of the phylum Epsilonbacteraeota on control beads compared with other bead types at day 35 supports this conclusion (Fig. 3).

## Conclusion

The use of nylon mesh pouches for the incubation of model particles in *in situ* conditions has allowed us to characterize the particle‐attaching community in the Black Sea avoiding the potential problems associated with laboratory bottle incubations. The particle‐attaching microbial community was shown to be remarkably similar to other marine environments, showing that microbial community members of specific niches can be recruited from highly different marine environments (i.e. from oxic to sulfidic systems) and confirming that particle attachment is a phylogenetically conserved trait. In addition, variation across the bead types points to the ability of microbes to recognize specific organic substrates, and indicates that the community is readily induced and selected by the availability of labile organic compounds also in the deep sulfidic water column.

To further test the succession of degradation processes and microbial communities, we also conducted laboratory bottle incubations under anoxic conditions. These incubations show that microorganisms highly specific for substrates readily available in the marine environment can be recruited, though the degradation of OM provided did not end up being the defining factor in most cases in our model particle system. Instead, a chemoautotrophic community defined because of sulfide concentrations was induced with time, indicating that the particles act as a surface for colonization and biofilm formation rather than for substrate degradation. Sulfurization of OM in sulfidic water columns has been detected and could also render the OM unavailable to microbial degradation (Raven *et al*., 2016), as well as mineral matrices that shield OM from microbial degradation (Hedges *et al*., 2001). The incubation conditions could have played a part in inducing this community, but the previous detection of similar organisms in the Black Sea and elsewhere on particles (Grote *et al*., 2008; Fuchsman *et al*., 2011; Fontanez *et al*., 2015; Boeuf *et al*., 2019) indicates that these communities take advantage of particles also in natural conditions. In addition, the evidence of chemoautotrophy detected in Black Sea SPM (Karl *et al*., 1984; Wakeham and Beier, 1991; Çoban‐Yildiz *et al*., 2006; Yilmaz *et al*., 2006) suggests that natural particles below the chemocline in the Black Sea are possibly not sites of intense heterotrophic activity but rather a convenient surface for biofilm formation allowing close interactions between chemoautotrophic organisms that are responsible for driving diverse biogeochemical cycles. These results provide support for chemoautotrophic carbon fixation as a possible factor in reducing the particle attenuation coefficient in oxygen‐depleted water columns (Keil *et al*., 2016). Longer incubation periods *in situ* with for example pure particle forms of simple substrates in connection with rate measurements would help answer these remaining questions on the extent of OM degradation capabilities in highly reduced environments.

## Experimental procedures

### Sampling

Sample water was collected at a station in the western Black Sea located at 42° 53.8′ N 30° 40.7′ E during a cruise with the research vessel R/V Pelagia in August 2018. Water column profiling was conducted using an ultra‐clean conductivity‐temperature‐density (CTD) system, which was equipped with among others a SBE3plus thermometer, SBE4 conductivity sensor and SBE43 dissolved oxygen sensor (Sea‐Bird Electronics, Bellevue, WA). Water samples used for bottle incubations were obtained using clean 25 l bottles that were connected to N_2_‐gas immediately after boarding of the sampler to minimize contact with oxygen. To analyse the total water column microbial community composition, SPM samples were collected with *in situ* pumps (McLane Laboratories, Falmouth, MA), by filtering through 142‐mm‐diameter 0.3 μm pore‐size glass fibre GF75 filters (Advantec MFS., Dublin, CA) and immediately stored at −80°C. While this filter size will potentially miss ultrasmall prokaryotic cells, it is in the range of filter sizes generally used to collect free‐living cells (0.2 μm) compared with the filter sized used to collect particle‐attached microbial communities (2.7 μm/30 μm; Fuchsman *et al*., 2011, Suter *et al*. 2018), and therefore approximates the total microbial community residing in the water column.

### Beads used for incubation experiments

To simulate POM with a different chemical composition, incubations were performed using magnetic beads of two major types. Either with substrates directly attached to the magnetic core, or beads with an agarose matrix, to which the substrate was covalently bonded. Magnetic beads were purchased as commercially available stocks. Pure chitin and protein A (New England Biolabs, USA) was directly attached to the core, while the agarose beads were coated with three different carbon sources: peptidoglycan, cellulose and hexadecanoic acid (Cube Biotech GmbH, Germany). Uncoated agarose beads were used as a control. The chitin‐coated beads had a diameter of 100 μm, while all other beads had a diameter of 30 μm, except for the protein A beads, which had on average 2 μm diameter.

Purchased bead solutions were washed 3× times with sterile basal salt medium (BSM) and diluted into anoxic BSM to make intermediate stock solutions. The BSM contained 4.4 mM of NaCl, 24.6 mM of MgCL_2_·6H_2_O, 9.5 mM of CaCl_2_ and 6.7 mM of KCl. Chitin and protein bead solutions were subsequently diluted 10×, while the other type of beads were diluted 80× to make the final anoxic bead stock solutions.

### In situ incubations

*In situ* incubations were performed with custom‐made nylon mesh pouches (Fig. S2). Nylon fabric with a mesh size of 20 μm (Solana, Belgium) was folded into a pouch of 4 × 7 cm^2^ dimensions. The pouches were filled with 50 μl of the originally purchased chitin bead stock or 20 μl of cellulose, peptidoglycan, hexadecanoic acid and uncoated beads. Each pouch had two compartments separated by the fabric, which were treated as duplicates. The seams of the pouches were heat‐sealed, and subsequently the pouches were attached to a fish line and placed inside a bottle on the CTD frame prior to *in situ* incubation. The bottles were incubated at 1000 and 2000 m for 6 h, before the bottles were closed and recovered on deck. The CTD bottles were open while they were lowered to these depths, bringing the beads in contact with the total water column. However, we consider that due to the speed of lowering (~1 m s^−1^), the low fraction of the total time spent in this, and the more extreme conditions in the deep water column, it is unlikely that microorganisms would have attached to the beads during the deployment of the bottles. After recovery, the pouches were immediately placed at 8°C, cut open and their content was resuspended in 20 ml BSM. After resuspension, the beads were collected with a magnet, the BSM was discarded and replaced with 1 ml BSM, and these samples were subsequently stored at −80°C for subsequent analysis.

### Bottle incubations

Glass pressure bottles of 1 L were first acid‐washed, autoclaved, and filled with N_2_. Seventy two bottles were filled with approximately 500 ml of seawater from different depths (100, 1000 and 2000 m), and pressurized to approximately 1.5 bar with N_2_ gas. Stock solutions of beads were added to these bottles. Each experiment was performed in triplicate. The larger chitin beads were added to the pressured bottles containing anoxic seawater to a final concentration of approximately 100 beads ml^−1^, while the agarose bead types were diluted to 400 beads ml^−1^. The final concentration of protein beads was not determined. Bottles were incubated in the dark at 8°C and were shaken to mix approximately every second day. After incubation for 7 days, 100 ml of sample water with beads was harvested from the bottles with a sterile N_2_ flushed needle and syringe. Water was collected twice in a 50 ml sterile falcon tube with a magnet attached that collected the magnetic beads to concentrate the number of beads two times. The remaining water was collected for nutrient analysis. The remaining magnetic beads were resuspended in 1 ml BSM, collected in an Eppendorf tube (2 ml) and placed in a magnetic rack. Another 900 μl of water was removed from the sample to concentrate the beads 20 times in total. The remaining 100 μl was stored at −80°C for subsequent analysis. Sample bottles were transported between the sampling points 7 and 35 days from the research vessel to the NIOZ laboratory, in the dark and at constant temperature.

### Nutrient and DIC analysis

Water column samples as well as remaining water from incubation was filtered through a 0.2 μm polycarbonate Whatman syringe filter (GE Healthcare Europe GmbH, Germany) and stored for nutrient analyses. For inorganic nitrogen concentrations approximately 3 ml was collected in a pony vial and stored at −20°C. Concentrations of NO_3_^−^ and NO_2_^−^ (Grasshoff, 1983) and NH_4_^+^ (Helder and De Vries, 1979) were analysed using TRAACS Gas Segmented Continuous Flow Analyser (Seal Analytical, UK). All measurements were calibrated with standards diluted in low nutrient seawater in the salinity range of the samples at approximately 22‰ to ensure that analysis remained within the same ionic strength. The instrument was calibrated with a standard addition curve of potassium phthalate (0; 25; 50; 100; 200 μmol C L^−1^). DIC concentrations were measured with the method of Stol *et al*. (2001).

### DNA extraction

Genomic DNA was extracted using a MasterPure kit (Epicentre, USA) from the concentrated bead samples. After defrosting, cells attached to sampled beads were lysed using a buffer containing 10 mM Tris–HCL and 1 mM EDTA and 5 μl of 50 mg ml^−1^ lysozyme and incubating for 30 min at 37°C, after which the extraction protocol was followed. DNA was precipitated by incubation of the samples with glycogen for 30 min at −20°C prior to centrifugation and washing. DNA from 1/32 of the SPM filters was extracted using the Powersoil® DNA Isolation kit according to the manufacturer's instructions (MoBio, Qiagen, Carlsbad, CA), except with an elution step with 50 μl TE. DNA concentrations were measured fluorometrically with Qubit™ dsDNA HS assay kit (Thermo Fischer Scientific, Waltham, MA), and extracts were stored at −80°C.

### 16S rRNA gene amplicon sequencing and analysis

16S rRNA amplicon sequencing was used to assess the microbial community composition of the bead associated bacteria. PCR amplicons were made in triplicates using Phusion High‐Fidelity *Taq* polymerase (Thermo Fischer Scientific) in Phusion HF buffer, with 0.2 mM dNTPs, 800 μg ml^−1^ BSA and 0.6 μM barcoded primers 515F‐Y and the reverse primer 806RB (Caporaso *et al*., 2011; Apprill *et al*., 2015; Parada *et al*., 2016). Primers targeting the 16s rRNA V4 hypervariable region fragment with a length of approximately 300 bp were used. rRNA was amplified using the following cycling conditions: initial denaturation at 98°C for 30 s followed by 30 cycles of: denaturation at 98°C for 10 s, primer annealing at 50°C for 20 s and primer extension at 72°C for 30 s. Triplicate PCR products were run on a 1% agarose for 50 min at 75 V. After running, the bands were carefully cut out and the triplicates were pooled. The PCR product was extracted from the gel using a QIAquick Gel Extraction Kit and accessory protocol (Qiagen). DNA concentration was measured using Qubit 4 Fluorometer with dsDNA HS Assay Kit (Thermo Fisher Scientific). Samples were diluted to 2 ng μl^−1^ and pooled before sequencing. Sequencing was performed by the Utrecht Sequencing Facility (Utrecht, the Netherlands), using an Illumina MiSeq sequencing platform. The Cascabel pipeline was used for the analysis of the 16S rRNA gene amplicon sequences (Asbun *et al*., 2019). This included quality assessment by FastQC (v. 0.11.3, Andrews, 2010), assembly of the paired‐end reads with Pear (v. 0.9.8, Zhang *et al*., 2013), with a minimum length of reads of 20, minimum overlap set at 7 and a *p*‐value threshold of 0.05. OTUs were picked with uclust with a 97% threshold and the longest sequence of each OTU cluster was picked as representative using QIIME (v. 1.9.1, Caporaso *et al*., 2010). The taxonomy of the representative sequences was assigned using uclust (75% cut‐off) against the SILVA database release 132 (Quast *et al*., 2012; https://www.arb-silva.de/). Samples with less than 16 000 OTUs and OTUs with an abundance of less than 0.02% were removed. Further analysis of the data was performed in R using the Phyloseq (v. 1.26.0, McMurdie and Holmes, 2013) and ampvis2 (v. 2.4.2, Andersen *et al*., 2018) packages. The 16S rRNA gene amplicon reads (raw data) have been deposited in the NCBI Sequence Read Archive under the BioProject ID PRJNA622458.

### Statistical analysis

Bray–Curtis dissimilarity was used to calculate the dissimilarity between samples in R. Statistical significance between the different sample groups were tested using PERMANOVA with the Adonis function available in the vegan package (v. 2.5.3, Oksanen *et al*., 2018). A NMDS was used to find the axes of most dissimilarity using the ordination function. To analyse the differentially abundant taxonomic groups across treatments we used the Deseq2 analysis of log_2_ fold changes in R (v. 1.20.0, Love *et al*., 2014). Deseq2 is a method used to analyse the differential abundance of sequencing count data between samples. It uses appropriate methods for the normalization and variance modelling of counts resulting from next‐generation sequencing platforms. While it has been developed for the analysis of differentially expressed RNA data, it can also be used to analyse differential abundance in OTU count data (McMurdie and Holmes, 2014). Briefly, in this method significant differences in abundance are analysed by modelling fold change using logistic regression with the negative binomial as family, and determining significance with a Wald test and Benjamini–Hochberg correction for multiple testing. Sequencing count data are modelled using the negative binomial distribution because of its specific characteristics. These include the low probability of pulling out a specific sequence from the large amount of possibilities and the overdispersion caused by the variance increasing with the mean. The false discovery rate was set at 0.01.

## Supporting information

**Supplementary Fig. S1.** Nitrate, nitrite and DIC concentrations during sampling times across all samples.
**Supplementary Fig. S2.** Photograph of *in situ* pouches A. before and B. after deployment.
**Supplementary Fig. S3.** Raw counts of selected OTUs in protein beads at 7 days incubation.
**Supplementary Fig. S4.** Physical water column CTD measurements at sampling.
**Supplementary Table. S1.** Results of PERMANOVA analysis of variance across different depths and time points.

## References

[emi15024-bib-0001] Alldredge, A.L., and Jackson, G.A. (1995) Aggregation in marine systems. Deep‐Sea Res 42: 1–8.

[emi15024-bib-0002] Alldredge, A.L., and Silver, M.W. (1988) Characteristics, dynamics and significance of marine snow. Prog Oceanogr 20: 41–82.

[emi15024-bib-0003] Alonso, C., and Pernthaler, J. (2005) Incorporation of glucose under anoxic conditions by bacterioplankton from coastal north sea surface waters. Appl Environ Microbiol 71: 1709–1716. 15811993 10.1128/AEM.71.4.1709-1716.2005 PMC1082556

[emi15024-bib-0004] Andersen, K.S., Kirkegaard, R.H., Karst, S.M., and Albertsen, M. (2018) ampvis2: an R package to analyse and visualise 16S rRNA amplicon data.

[emi15024-bib-0005] Andrews S. (2010) FastQC: a quality control tool for high throughput sequence data. Available online at: http://www.bioinformatics.babraham.ac.uk/projects/fastqc

[emi15024-bib-0006] Apprill, A., McNally, S., Parsons, R., and Weber, L. (2015) Minor revision to V4 region SSU rRNA 806R gene primer greatly increases detection of SAR11 bacterioplankton. Aquat Microb Ecol 75: 129–137.

[emi15024-bib-0007] Arnosti, C., Repeta, D.J., and Blough, N.V. (1994) Rapid bacterial degradation of polysaccharides in anoxic marine systems. Geochim Cosmochim Acta 58: 2639–2652.

[emi15024-bib-0008] Asbun, A.A., Besseling, M.A., Balzano, S., van Bleijswijk, J., Witte, H., Villanueva, L., and Engelmann, J.C. (2019) Cascabel: a flexible, scalable and easy‐to‐use amplicon sequence data analysis pipeline. bioRxiv 809384.

[emi15024-bib-0009] Azam, F., and Ammerman, J.W. (1984) Mechanisms of organic matter utilization by marine bacterioplankton. In Marine Phytoplankton and Productivity, Holm‐Hansen, O., Bolis, L., and Gilles, R. (eds). Berlin: Springer‐Verlag, pp. 45–54.

[emi15024-bib-0010] Azam, F., and Malfatti, F. (2007) Microbial structuring of marine ecosystems. Nat Rev Microbiol 5: 782–791. 17853906 10.1038/nrmicro1747

[emi15024-bib-0011] Bachmann, J., Heimbach, T., Hassenrück, C., Kopprio, G.A., Iversen, M.H., Grossart, H.P., and Gärdes, A. (2018) Environmental drivers of free‐living vs. particle‐attached bacterial community composition in the Mauritania upwelling system. Front Microbiol 9: 1–13. 30532746 10.3389/fmicb.2018.02836 PMC6265507

[emi15024-bib-0012] Beier, S., and Bertilsson, S. (2013) Bacterial chitin degradation‐mechanisms and ecophysiological strategies. Front Microbiol 4: 1–12. 23785358 10.3389/fmicb.2013.00149 PMC3682446

[emi15024-bib-0013] Boeuf, D., Edwards, B.R., Eppley, J.M., Hu, S.K., Poff, K.E., Romano, A.E., *et al*. (2019) Biological composition and microbial dynamics of sinking particulate organic matter at abyssal depths in the oligotrophic open ocean. Proc Natl Acad Sci U S A 116: 11824–11832. 31127042 10.1073/pnas.1903080116 PMC6575173

[emi15024-bib-0095] Caporaso, J.G., Lauber, C.L., Walters, W.A., Berg‐Lyons, D., Lozupone, C.A., Turnbaugh, P.J., et al. (2011) Global patterns of 16S rRNA diversity at a depth of millions of sequences per sample. Proc Natl Acad Sci U S A 108: 4516–4522. 20534432 10.1073/pnas.1000080107 PMC3063599

[emi15024-bib-0014] Bruckner, C.G., Mammitzsch, K., Jost, G., Wendt, J., Labrenz, M., and Jürgens, K. (2013) Chemolithoautotrophic denitrification of epsilonproteobacteria in marine pelagic redox gradients. Environ Microbiol 15: 1505–1513. 23013279 10.1111/j.1462-2920.2012.02880.x

[emi15024-bib-0015] Burdige, D.J. (2007) Preservation of organic matter in marine sediments: controls, mechanisms, and an imbalance in sediment organic carbon budgets? Chem Rev 107: 467–485. 17249736 10.1021/cr050347q

[emi15024-bib-0016] Byrne, J.M., Van Der Laan, G., Figueroa, A.I., Qafoku, O., Wang, C., Pearce, C.I., *et al*. (2016) Size dependent microbial oxidation and reduction of magnetite nano‐and micro‐particles. Sci Rep 6: 1–13. 27492680 10.1038/srep30969 PMC4974511

[emi15024-bib-0017] Campbell, B.J., Engel, A.S., Porter, M.L., and Takai, K. (2006) The versatile ε‐proteobacteria: key players in sulphidic habitats. Nat Rev Microbiol 4: 458–468. 16652138 10.1038/nrmicro1414

[emi15024-bib-0018] Canfield, D.E., and Berner, R.A. (1987) Dissolution and pyritization of magnetite in anoxie marine sediments. Geochim Cosmochim Acta 51: 645–659.

[emi15024-bib-0019] Caporaso, J.G., Kuczynski, J., Stombaugh, J., Bittinger, K., Bushman, F.D., Costello, E.K., *et al*. (2010) QIIME allows analysis of high‐throughput community sequencing data. Nat Methods 7: 335–336. 20383131 10.1038/nmeth.f.303 PMC3156573

[emi15024-bib-0020] Castelle, C.J., Brown, C.T., Anantharaman, K., Probst, A.J., Huang, R.H., and Banfield, J.F. (2018) Biosynthetic capacity, metabolic variety and unusual biology in the CPR and DPANN radiations. Nat Rev Microbiol 16: 629–645. 30181663 10.1038/s41579-018-0076-2

[emi15024-bib-0021] Cavan, E.L., Trimmer, M., Shelley, F., and Sanders, R. (2017) Remineralization of particulate organic carbon in an ocean oxygen minimum zone. Nat Commun 8: 14847. 28322218 10.1038/ncomms14847 PMC5364423

[emi15024-bib-0022] Çoban‐Yildiz, Y., Altabet, M.A., Yilmaz, A., and Tuǧrul, S. (2006) Carbon and nitrogen isotopic ratios of suspended particulate organic matter (SPOM) in the Black Sea water column. Deep Res Part II Top Stud Oceanogr 53: 1875–1892.

[emi15024-bib-0023] Datta, M.S., Sliwerska, E., Gore, J., Polz, M., and Cordero, O.X. (2016) Microbial interactions lead to rapid micro‐scale successions on model marine particles. Nat Commun 7: 11965. 27311813 10.1038/ncomms11965 PMC4915023

[emi15024-bib-0024] Dellwig, O., Leipe, T., März, C., Glockzin, M., Pollehne, F., Schnetger, B., *et al*. (2010) A new particulate Mn‐Fe‐P‐shuttle at the redoxcline of anoxic basins. Geochim Cosmochim Acta 74: 7100–7115.

[emi15024-bib-0025] DeLong, E.F., Franks, D.G., and Alldredge, A.L. (1993) Phylogenetic diversity of aggregate‐attached marine bacterial assemblages. Limnol Oceanogr 38: 924–934.

[emi15024-bib-0026] Devol, A.H., and Hartnett, H.E. (2001) Role of the oxygen‐deficient zone in transfer of organic carbon to the deep ocean. Limnol Oceanogr 46: 1684–1690.

[emi15024-bib-0027] Doghri, I., Rodrigues, S., Bazire, A., Dufour, A., Akbar, D., Sopena, V., *et al*. (2015) Marine bacteria from the French Atlantic coast displaying high forming‐biofilm abilities and different biofilm 3D architectures. BMC Microbiol 15: 1–10. 26498445 10.1186/s12866-015-0568-4 PMC4619314

[emi15024-bib-0028] Engel, A., Wagner, H., Le Moigne, F.A.C., and Wilson, S.T. (2017) Particle export fluxes to the oxygen minimum zone of the eastern tropical North Atlantic. Biogeosciences 14: 1825–1838.

[emi15024-bib-0029] Enke, T.N., Datta, M.S., Schwartzman, J., Cermak, N., Schmitz, D., Barrere, J., *et al*. (2019) Modular assembly of polysaccharide‐degrading marine microbial communities. Curr Biol 29: 1528–1535.e6. 31031118 10.1016/j.cub.2019.03.047

[emi15024-bib-0030] Fontanez, K.M., Eppley, J.M., Samo, T.J., Karl, D.M., and DeLong, E.F. (2015) Microbial community structure and function on sinking particles in the North Pacific Subtropical Gyre. Front Microbiol 6: 1–14. 26042105 10.3389/fmicb.2015.00469 PMC4436931

[emi15024-bib-0031] Fuchsman, C.A., Kirkpatrick, J.B., Brazelton, W.J., Murray, J.W., and Staley, J.T. (2011) Metabolic strategies of free‐living and aggregate‐associated bacterial communities inferred from biologic and chemical profiles in the Black Sea suboxic zone. FEMS Microbiol Ecol 78: 586–603. 22066565 10.1111/j.1574-6941.2011.01189.x

[emi15024-bib-0032] Fuchsman, C.A., Murray, J.W., and Staley, J.T. (2012b) Stimulation of autotrophic denitrification by intrusions of the Bosporus Plume into the anoxic Black Sea. Front Microbiol 3: 1–14. 22826706 10.3389/fmicb.2012.00257 PMC3399223

[emi15024-bib-0033] Fuchsman, C.A., Paul, B., Staley, J.T., Yakushev, E.V., and Murray, J.W. (2019) Detection of transient denitrification during a high organic matter event in the Black Sea. Global Biogeochem Cycles 33: 143–162.

[emi15024-bib-0034] Fuchsman, C.A., Staley, J.T., Oakley, B.B., Kirkpatrick, J.B., and Murray, J.W. (2012a) Free‐living and aggregate‐associated Planctomycetes in the Black Sea. FEMS Microbiol Ecol 80: 402–416. 22251018 10.1111/j.1574-6941.2012.01306.x

[emi15024-bib-0035] Ganesh, S., Parris, D.J., DeLong, E.F., and Stewart, F.J. (2014) Metagenomic analysis of size‐fractionated picoplankton in a marine oxygen minimum zone. ISME J 8: 187–211. 24030599 10.1038/ismej.2013.144 PMC3869020

[emi15024-bib-0036] Grasshoff, K. (1983) Methods of Seawater Analysis. Weinheim: Verlag Chemie.

[emi15024-bib-0037] Grossart, H.P., Kiørboe, T., Tang, K.W., Allgaier, M., Yam, E.M., and Ploug, H. (2006) Interactions between marine snow and heterotrophic bacteria: aggregate formation and microbial dynamics. Aquat Microbial Ecol 42: 19–26.

[emi15024-bib-0038] Grote, J., Jost, G., Labrenz, M., Herndl, G.J., and Jürgens, K. (2008) Epsilonproteobacteria represent the major portion of chemoautotrophic bacteria in sulfidic waters of pelagic redoxclines of the Baltic and black seas. Appl Environ Microbiol 74: 7546–7551. 18952879 10.1128/AEM.01186-08 PMC2607176

[emi15024-bib-0039] Gulmann, L.K., Beaulieu, S.E., Shank, T.M., Ding, K., Seyfried, W.E., and Sievert, S.M. (2015) Bacterial diversity and successional patterns during biofilm formation on freshly exposed basalt surfaces at diffuse‐flow deep‐sea vents. Front Microbiol 6: 1–16. 26441852 10.3389/fmicb.2015.00901 PMC4564720

[emi15024-bib-0040] Harvey, H.R., Tuttle, J.H., and Tyler Bell, J. (1995) Kinetics of phytoplankton decay during simulated sedimentation: changes in biochemical composition and microbial activity under oxic and anoxic conditions. Geochim Cosmochim Acta 59: 3367–3377.

[emi15024-bib-0041] Hedges, J.I., Baldock, J.A., Gelinas, Y., Lee, C., Peterson, M., and Wakeham, S.G. (2001) Evidence for non‐selective preservation of organic matter in sinking marine particles. Nature 409: 801–804. 11236989 10.1038/35057247

[emi15024-bib-0042] Helder, W., and de Vries, R.P.T. (1979) An automatic phenolhypochlorite method for the determination of ammonia in sea‐ and brackish waters. Neth J Sea Res 13: 154–160.

[emi15024-bib-0043] Henkel, J.V., Dellwig, O., Pollehne, F., Herlemann, D.P.R., Leipe, T., and Schulz‐Vogt, H.N. (2019) A bacterial isolate from the Black Sea oxidizes sulfide with manganese(IV) oxide. Proc Natl Acad Sci U S A 116: 12153–12155. 31160458 10.1073/pnas.1906000116 PMC6589767

[emi15024-bib-0044] Herndl, G.J., and Reinthaler, T. (2013) Microbial control of the dark end of the biological pump. Nat Geosci 6: 718–724. 24707320 10.1038/ngeo1921 PMC3972885

[emi15024-bib-0045] Jeuniaux, C., and Voss‐Foucart, M.F. (1991) Chitin biomass and production in the marine environment. Biochem Syst Ecol 19: 347–356.

[emi15024-bib-0046] Ji, S., Wang, S., Tan, Y., Chen, X., Schwarz, W., and Li, F. (2012) An untapped bacterial cellulolytic community enriched from coastal marine sediment under anaerobic and thermophilic conditions. FEMS Microbiol Lett 335: 39–46. 22788522 10.1111/j.1574-6968.2012.02636.x

[emi15024-bib-0047] Jiao, N., Herndl, G.J., Hansell, D.a., Benner, R., Kattner, G., Wilhelm, S.W., *et al*. (2010) Microbial production of recalcitrant dissolved organic matter: long‐term carbon storage in the global ocean. Nat Rev Microbiol 8: 593–599. 20601964 10.1038/nrmicro2386

[emi15024-bib-0048] Kalvelage, T., Lavik, G., Lam, P., Contreras, S., Arteaga, L., Löscher, C.R., *et al*. (2013) Nitrogen cycling driven by organic matter export in the South Pacific oxygen minimum zone. Nat Geosci 6: 228–234.

[emi15024-bib-0049] Karl, D.M., and Knauer, G.A. (1991) Microbial production and particle flux in the upper 350 m of the Black Sea. Deep Sea Res Part A Oceanogr Res Pap 38: S921–S942.

[emi15024-bib-0050] Karl, D.M., Knauer, G.A., Martin, J.H., and Ward, B.B. (1984) Bacterial chemolithotrophy in the ocean is associated with sinking particles. Nature 309: 54–56.

[emi15024-bib-0051] Keil, R.G., Neibauer, J.A., Biladeau, C., Van Der Elst, K., and Devol, A.H. (2016) A multiproxy approach to understanding the “enhanced” flux of organic matter through the oxygen‐deficient waters of the Arabian Sea. Biogeosciences 13: 2077–2092.

[emi15024-bib-0052] Kok, M.D., Schouten, S., and Sinninghe Damsté, J.S. (2000) Formation of insoluble, nonhydrolyzable, sulfur‐rich macromolecules via incorporation of inorganic sulfur species into algal carbohydrates. Geochim Cosmochim Acta 64: 2689–2699.

[emi15024-bib-0053] Lee, J.W., Nam, J.H., Kim, Y.H., Lee, K.H., and Lee, D.H. (2008) Bacterial communities in the initial stage of marine biofilm formation on artificial surfaces. J Microbiol 46: 174–182. 18545967 10.1007/s12275-008-0032-3

[emi15024-bib-0054] Liu, X., Li, M., Castelle, C.J., Probst, A.J., Zhou, Z., Pan, J., *et al*. (2018) Insights into the ecology, evolution, and metabolism of the widespread Woesearchaeotal lineages. Microbiome 6: 102. 29884244 10.1186/s40168-018-0488-2 PMC5994134

[emi15024-bib-0055] Love, M.I., Huber, W., and Anders, S. (2014) Moderated estimation of fold change and dispersion for RNA‐seq data with DESeq2. Gen Biol 15: 550. 10.1186/s13059-014-0550-8 PMC4302049 25516281

[emi15024-bib-0056] Malvankar, N.S., King, G.M., and Lovley, D.R. (2015) Centimeter‐long electron transport in marine sediments via conductive minerals. ISME J 9: 527–531. 25050525 10.1038/ismej.2014.131 PMC4303629

[emi15024-bib-0057] McMurdie, P.J., and Holmes, S. (2013) Phyloseq: an R package for reproducible interactive analysis and graphics of microbiome census data. PLoS ONE 8: e61217. 23630581 10.1371/journal.pone.0061217 PMC3632530

[emi15024-bib-0058] McMurdie, P.J., and Holmes, S. (2014) Waste not, want not: why rarefying microbiome data is inadmissible. PLoS Comput Biol 10: e1003531. 24699258 10.1371/journal.pcbi.1003531 PMC3974642

[emi15024-bib-0059] Mestre, M., Ferrera, I., Borrull, E., Ortega‐Retuerta, E., Mbedi, S., Grossart, H.P., *et al*. (2017) Spatial variability of marine bacterial and archaeal communities along the particulate matter continuum. Mol Ecol 26: 6827–6840. 29117634 10.1111/mec.14421

[emi15024-bib-0060] Nelson, W.C., and Stegen, J.C. (2015) The reduced genomes of Parcubacteria (OD1) contain signatures of a symbiotic lifestyle. Front Microbiol 6: 1–14. 26257709 10.3389/fmicb.2015.00713 PMC4508563

[emi15024-bib-0061] Oksanen, J., Blanchet, F.G., Friendly, M., Kindt, R., Legendre, P., McGlinn, D. *et al*. (2018) vegan: Community Ecology Package. R package version 2.5–3. https://CRAN.R‐ project.org/package=vegan

[emi15024-bib-0062] Orsi, W.D., Smith, J.M., Liu, S., Liu, Z., Sakamoto, C.M., Wilken, S., *et al*. (2016) Diverse, uncultivated bacteria and archaea underlying the cycling of dissolved protein in the ocean. ISME J 10: 2158–2173. 26953597 10.1038/ismej.2016.20 PMC4989311

[emi15024-bib-0063] Parada, A.E., Needham, D.M., and Fuhrman, J.A. (2016) Every base matters: assessing small subunit rRNA primers for marine microbiomes with mock communities, time series and global field samples. Environ Microbiol 18: 1403–1414. 26271760 10.1111/1462-2920.13023

[emi15024-bib-0064] Quast, C., Pruesse, E., Yilmaz, P., Gerken, J., Schweer, T., Yarza, P., *et al*. (2012) The SILVA ribosomal RNA gene database project: improved data processing and web‐based tools. Nucleic Acids Res 41: D590–D596. 23193283 10.1093/nar/gks1219 PMC3531112

[emi15024-bib-0065] Rahman, N.A., Parks, D.H., Vanwonterghem, I., Morrison, M., Tyson, G.W., and Hugenholtz, P. (2016) A phylogenomic analysis of the bacterial phylum fibrobacteres. Front Microbiol 6: 1469. 26779135 10.3389/fmicb.2015.01469 PMC4704652

[emi15024-bib-0066] Raven, M.R., Sessions, A.L., Adkins, J.F., and Thunell, R.C. (2016) Rapid organic matter sulfurization in sinking particles from the Cariaco Basin water column. Geochim Cosmochim Acta 190: 175–190.

[emi15024-bib-0067] Roullier, F., Berline, L., Guidi, L., Durrieu De Madron, X., Picheral, M., Sciandra, A., *et al*. (2014) Particle size distribution and estimated carbon flux across the Arabian Sea oxygen minimum zone. Biogeosciences 11: 4541–4557.

[emi15024-bib-0068] Salazar, G., Cornejo‐Castillo, F.M., Borrull, E., Díez‐Vives, C., Lara, E., Vaqué, D., *et al*. (2015) Particle‐association lifestyle is a phylogenetically conserved trait in bathypelagic prokaryotes. Mol Ecol 24: 5692–5706. 26462173 10.1111/mec.13419

[emi15024-bib-0069] Schulz‐Vogt, H.N., Pollehne, F., Jürgens, K., Arz, H.W., Beier, S., Bahlo, R., *et al*. (2019) Effect of large magnetotactic bacteria with polyphosphate inclusions on the phosphate profile of the suboxic zone in the Black Sea. ISME J 13: 1198–1208. 30643197 10.1038/s41396-018-0315-6 PMC6474215

[emi15024-bib-0070] Schwarz, W.H. (2001) The cellulosome and cellulose degradation by anaerobic bacteria. Appl Microbiol Biotechnol 56: 634–649. 11601609 10.1007/s002530100710

[emi15024-bib-0071] Silver, M.W., Shanks, A.L., and Trent, J.D. (1978) Marine snow: microplankton habitat and source of small‐scale patchiness in pelagic populations. Science 201: 371–373. 17793735 10.1126/science.201.4353.371

[emi15024-bib-0072] Sinninghe Damsté, J.S., and de Leeuw, J.W. (1990) Analysis, structure and geochemical significance of organically‐bound sulfur in the geosphere: state of the art and future research. Org Geochem 16: 1077–1101.

[emi15024-bib-0073] Sinninghe Damsté, J.S., Rijpstra, W.I.C., Coolen, M.J.L., Schouten, S., and Volkman, J.K. (2007) Rapid sulfurisation of highly branched isoprenoid (HBI) alkenes in sulfidic Holocene sediments from Ellis Fjord, Antarctica. Org Geochem 38: 128–139.

[emi15024-bib-0074] Smith, M.W., Allen, L.Z., Allen, A.E., Herfort, L., and Simon, H.M. (2013) Contrasting genomic properties of free‐living and particle‐attached microbial assemblages within a coastal ecosystem. Front Microbiol 4: 1–20. 23750156 10.3389/fmicb.2013.00120 PMC3668451

[emi15024-bib-0075] Sollai, M., Villanueva, L., Hopmans, E.C., Reichart, G.J., and Sinninghe Damsté, J.S. (2019) A combined lipidomic and 16S rRNA gene amplicon sequencing approach reveals archaeal sources of intact polar lipids in the stratified Black Sea water column. Geobiology 17: 91–109. 30281902 10.1111/gbi.12316 PMC6586073

[emi15024-bib-0076] Sorokin, D.Y., Gumerov, V.M., Rakitin, A.L., Beletsky, A.V., Damsté, J.S.S., Muyzer, G., *et al*. (2014) Genome analysis of Chitinivibrio alkaliphilus gen. nov., sp. nov., a novel extremely haloalkaliphilic anaerobic chitinolytic bacterium from the candidate phylum Termite Group 3. Environ Microbiol 16: 1549–1565. 24112708 10.1111/1462-2920.12284

[emi15024-bib-0077] Sorokin, Y.I., Sorokin, P.Y., Avdeev, V.A., Sorokin, D.Y., and Ilchenko, S.V. (1995) Biomass, production and activity of bacteria in the Black Sea, with special reference to chemosynthesis and the sulfur cycle. Hydrobiologia 308: 61–76.

[emi15024-bib-0078] Stewart, F.J., Dalsgaard, T., Young, C.R., Thamdrup, B., Revsbech, N.P., Ulloa, O., *et al*. (2012) Experimental incubations elicit profound changes in community transcription in OMZ bacterioplankton. PLoS One 7: e37118. 22615914 10.1371/journal.pone.0037118 PMC3353902

[emi15024-bib-0079] Stol, M.H.C., Bakker, K., Nobbe, G.H., and Hasse, R.R. (2001) Contiuous‐flow analysis of dissolved inorganic carbon content in seawater. Anal Chem 73: 4111–4116. 11569799 10.1021/ac010303r

[emi15024-bib-0094] Suominen, S., Dombrowski, N., Sinninghe Damsté, J.S., and Villanueva, L. (2020) A diverse uncultivated microbial community is responsible for organic matter degradation in the Black Sea sulphidic zone. Environmental Microbiology. 10.1111/1462-2920.14902 PMC8359207 31858660

[emi15024-bib-0081] Suter, E.A., Pachiadaki, M., Taylor, G.T., Astor, Y., and Edgcomb, V.P. (2018) Free‐living chemoautotrophic and particle‐attached heterotrophic prokaryotes dominate microbial assemblages along a pelagic redox gradient. Environ Microbiol 20: 693–712. 29160034 10.1111/1462-2920.13997

[emi15024-bib-0082] Taylor, G.T., Iabichella, M., Ho, T.Y., Scranton, M.I., Thunell, R.C., Muller‐Karger, F., and Varela, R. (2001) Chemoautotrophy in the redox transition zone of the Cariaco Basin: a significant midwater source of organic carbon production. Limnol Oceanogr 46: 148–163.

[emi15024-bib-0083] Torres‐Beltrán, M., Mueller, A., Scofield, M., Pachiadaki, M.G., Taylor, C., Tyshchenko, K., *et al*. (2019) Sampling and processing methods impact microbial community structure and potential activity in a seasonally anoxic fjord: Saanich inlet, British Columbia. Front Mar Sci 6: 1–16.

[emi15024-bib-0084] Van Mooy, B.A.S., Keil, R.G., and Devol, A.H. (2002) Impact of suboxia on sinking particulate organic carbon: enhanced carbon flux and preferential degradation of amino acids via denitrification. Geochim Cosmochim Acta 66: 457–465.

[emi15024-bib-0085] Vetriani, C., Tran, H.V., and Kerkhof, L.J. (2003) Fingerprinting microbial assemblages from the oxic/anoxic chemocline of the Black Sea. Appl Environ Microbiol 69: 6481–6488. 14602603 10.1128/AEM.69.11.6481-6488.2003 PMC262261

[emi15024-bib-0086] Volk, T., and Hoffert, M.I. (1985) Ocean carbon pumps: analysis of relative strengths and efficiencies in ocean ‐ driven atmospheric CO2 changes. In The Carbon Cycle and Atmospheric CO_2_: Natural Variations Archaean to Present, Sundquist, E.T., and Broecker, W. (eds). Washington, DC: American Geophysical Union, pp. 99–110.

[emi15024-bib-0087] Waite, D.W., Vanwonterghem, I., Rinke, C., Parks, D.H., Zhang, Y., Takai, K., *et al*. (2017) Comparative genomic analysis of the class Epsilonproteobacteria and proposed reclassification to epsilonbacteraeota (phyl. Nov.). Front Microbiol 8: 682. 28484436 10.3389/fmicb.2017.00682 PMC5401914

[emi15024-bib-0088] Wakeham, S.G., and Beier, J.A. (1991) Fatty acid and sterol biomarkers as indicators of particulate matter source and alteration processes in the Black Sea. Deep Res Part A 38: S943–S968.

[emi15024-bib-0089] Ward, B.B., Tuit, C.B., Jayakumar, A., Rich, J.J., Moffett, J., and Naqvi, S.W.A. (2008) Organic carbon, and not copper, controls denitrification in oxygen minimum zones of the ocean. Deep Res Part I Oceanogr Res Pap 55: 1672–1683.

[emi15024-bib-0090] Werne, J.P., Hollander, D.J., Behrens, A., Schaeffer, P., Albrecht, P., and Sinninghe Damsté, J.S. (2000) Timing of early diagenetic sulfurization of organic matter: a precursor‐product relationship in Holocene sediments of the anoxic Cariaco Basin, Venezuela. Geochim Cosmochim Acta 64: 1741–1751.

[emi15024-bib-0091] Yilmaz, A., Çoban‐Yildiz, Y., Telli‐Karakoç, F., and Bologa, A. (2006) Surface and mid‐water sources of organic carbon by photoautotrophic and chemoautotrophic production in the Black Sea. Deep Res Part II Top Stud Oceanogr 53: 1988–2004.

[emi15024-bib-0092] Zhang, J., Kobert, K., Flouri, T., and Stamatakis, A. (2013) PEAR: a fast and accurate Illumina paired‐end reAd mergeR. Bioinformatics 30: 614–620. 24142950 10.1093/bioinformatics/btt593 PMC3933873

[emi15024-bib-0093] Zhang, Y., Xiao, W., and Jiao, N. (2016) Linking biochemical properties of particles to particle‐attached and free‐living bacterial community structure along the particle density gradient from freshwater to open ocean. J Geophys Res Biogeo 121: 2261–2274.

