# Supplementary material for: Microbial community development on model particles in the deep sulfidic waters of the Black Sea
Source: Environ Microbiol. 2020 Apr 30;23(6):2729–46. doi: 10.1111/1462-2920.15024 (PMC8359284; doi:10.1111/1462-2920.15024)
Supplement: Supplementary file 1 — Supplementary Fig. S1. Nitrate, nitrite and DIC concentrations during sampling times across all samples. Supplementary Fig. S2. Photograph of in situ pouches A. before and B. after deployment. Supplementary Fig. S3. Raw counts of selected OTUs in protein beads at 7 days incubation. Supplementary Fig. S4. Physical water column CTD measurements at sampling. Supplementary Table. S1. Results of PERMANOVA analysis of variance across different depths and time points. [file EMI-23-2729-s001.docx]

Supplementary File for:

**Microbial community development on model particles in the deep sulfidic waters of the Black Sea**

Saara Suominen^1*^, Karlijn Doorenspleet^1^, Jaap Sinninghe Damsté^1,2^, and Laura Villanueva^1^

^1^Department of Marine Microbiology and Biogeochemistry, NIOZ Royal Netherlands Institute for Sea Research and Utrecht University, The Netherlands

^2^Department of Earth Sciences, Faculty of Geosciences, Utrecht University, The Netherlands.

* Corresponding author, [saara.suominen@nioz.nl](mailto:sigrid.van.grinsven@nioz.nl)

Postal address: NIOZ, PO Box 59, 1790 AB Den Burg (Texel), The Netherlands

Telephone & fax: Telephone: +31 (0)222 369 504 Fax: +31 (0)222 319 674

**Supplementary Figure 1,** Measurements of dissolved inorganic carbon (DIC), nitrate and nitrite at different sampling timepoints for the different depths. The boxplots show variation across all different bead types and replicates. Dashed lines represent values measured from the water column.


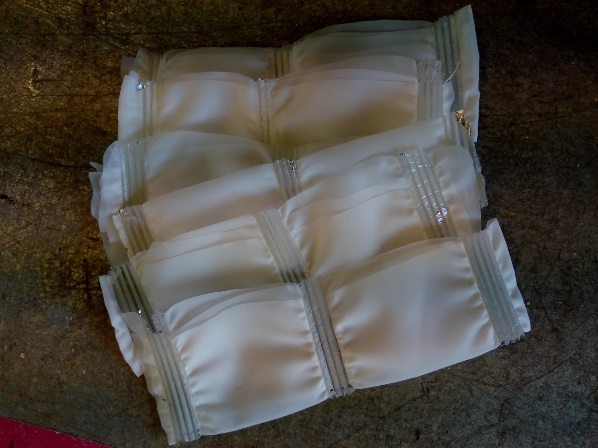


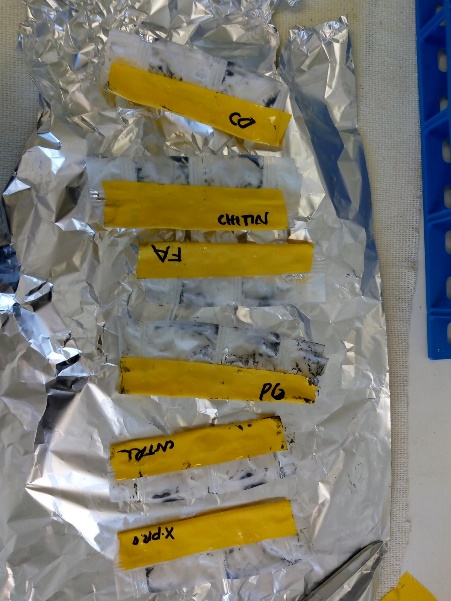


A

B

**Supplementary Figure S2,** Photograph of in situ pouches A. before and B. after deployment

**

**Supplementary Figure S3,** Examples of raw counts of OTUs that were determined significantly more abundant in protein bead incubations compared to control bead incubations at day 7

**Supplementary Figure S4,** Physicochemical measurements from CTD deployment across the water column at sampling site. Blue dots depict sampling points.

**Supplementary Table S1,** Permanova analysis of variance of different sample groups.

| All depths |  |  |  | **Day7** | | **Day35** | |
| --- | --- | --- | --- | --- | --- | --- | --- |
|  |  |  |  | *R2* | *p* | *R2* | *p* |
|  | **Type** |  |  | 0,18 | 0,001 | 0,17 | 0,001 |
|  | **Depth** |  |  | 0,08 | 0,001 | 0,07 | 0,001 |
|  | **Type*Depth** |  |  | 0,13 | 0,003 | 0,12 | 0,001 |
| Sulfidic depths |  | **In-situ** | | **Day7** | | **Day35** | |
|  |  | *R2* | *p* | *R2* | *p* | *R2* | *p* |
|  | **Type** | 0,25 | 0,001 | 0,34 | 0,001 | 0,28 | 0,001 |
|  | **Depth** | 0,10 | 0,002 | 0,04 | 0,15 | 0,04 | 0,001 |
|  | **Type*Depth** | 0,22 | 0,007 | 0,17 | 0,535 | 0,13 | 0,053 |
